# New Inventions

**Published:** 1897-10

**Authors:** 


### NEW INVENTIONS.

A hoof-cutting tool with stationary arm, having a head formed thereon, a sliding jaw having a rack thereon passing through said head, a swinging lever having a pinion and pivoted on the head for operating the sliding jaw, a vertical jaw extended from the head and having a lateral projection with a plate riveted thereto, an extension on said sliding jaw projected at an angle, a knife mounted thereon having its bevelled edge upon a horizontal line parallel with the plate of the projection on the fixed jaw, and also parallel with the rock-arm of the sliding jaw, the lower side of said extension inclining downward from the outer end, whereby the hoof may be cut without contact with the frog by the extension carrying the knife, and means for adjusting the knife in the extension.

A guiding device for bridles, comprising a ring to be placed between the cheek and lateral portion of the jaw, a ring also to be placed outside of the cheek more or less parallel with the other, and a stem integral with both rings, to bear rearwardly against an inner corner of the mouth.

A calk-sharpener and hoof-trimmer with a tool-holder, comprising a shank having tool-holding means, a box loosely encircling the shank, clamping members encircling the box, handles fitted in between the ends of the clamps, and bolts passing through the handles and clamp members.

A rein-fastening, comprising two metal plates, one being provided with a socket, the interior of which is wider on two sides than is its mouth, the other having a tongue provided with projections on opposite sides, and a spring in said socket, having its ends bent slightly in a direction toward each other, to engage the opposite projections of said tongue.

A bridle-bit, comprising a mouthpiece having a resilient metallic continuous strip for its core, the ends of which are provided with integral return-bends, a yielding strip at one side thereof having its ends secured between the core and its return-bend, whereby are formed closed loops for the rings, a yielding strip at the other side of the core, and a yielding covering for the core and strips.

